# Osteoarthritis: Can We Do Better?

**DOI:** 10.7759/cureus.31505

**Published:** 2022-11-14

**Authors:** Dylon P Collins, Kawther N Elsouri, Michelle Demory Beckler

**Affiliations:** 1 Osteopathic Medicine, Nova Southeastern University Dr. Kiran C. Patel College of Osteopathic Medicine, Fort Lauderdale, USA; 2 Microbiology and Immunology, Nova Southeastern University Dr. Kiran C. Patel College of Allopathic Medicine, Fort Lauderdale, USA

**Keywords:** streaming potentials, arthritis, joint imaging, cartilage degeneration, early osteoarthritis, electroarthrography, osteoarthritis

## Abstract

Osteoarthritis (OA) is the most common form of arthritis, affecting approximately 32.5 million adults in the United States. OA is characterized as a degenerative joint disease or “wear and tear” arthritis. Symptoms experienced by patients include, but are not limited to, swelling, stiffness, pain or aching, and decreased range of motion. The majority of individuals impacted by OA are over the age of 65. OA has long been thought of as an inevitable part of aging. Patients are typically diagnosed after the onset of symptoms once irreversible damage has occurred, such as the breakdown of bone and cartilage. Along with clinical presentations, medical professionals often relied on radiographic images to confirm the diagnosis of OA. Limited research has looked into how to catch OA early and stage patients in a pre-OA state, possibly preventing irreparable damage that is observable radiographically. This article presents the history, diagnosis, and classes of OA. In addition, we present multiple diagnostic tools currently used and others under investigation, including OA-specific biomarkers and electroarthrography (EAG). These tools show promise as aids in early OA diagnosis and intervention, ultimately slowing down or altogether stopping the progression of OA. In conjunction or individually, these techniques, if further developed, stand out as promising mechanisms that may decrease the current OA burden on the healthcare system.

## Introduction and background

Introduction

Estimates in the United States show that approximately 80% of the population, 65 years or older have radiologic findings of osteoarthritis (OA), but only 60% experience symptoms [1]. Successful early diagnosis and treatment may benefit the healthcare system by reducing long-term morbidity and strain on healthcare system resources [2]. Since some of the first publications on OA, healthcare providers have become better equipped to diagnose and manage the disease. However, their ability to intervene before irreversible joint changes has been limited. OA can occur within any joint but tends to occur within joints with high stresses, such as the hands, knees, hips, and spine [3]. Symptoms vary among patients based on their individual clinical manifestations. Pain is one of the main reasons patients consult their physician seeking relief. Age is the number one risk factor for developing OA. Other factors include female gender, previous injury to the joint, BMI, anatomical variance, and lifestyle [1]. The question remains on how to improve early detection methods and if current management protocols are doing enough to address the progression of OA.

History

OA is a progressively degenerative disease of the articular cartilage which provides protection and a cushion for smooth gliding between joint surfaces [3]. OA, rheumatoid arthritis (RA), and psoriatic arthritis were distinguished by Richard von Volkmann in the mid-1850s as the three major forms of arthritis, with osteoarthritis reintroduced and popularized by John Kent Spender in 1886 [4, 5]. In 1895, Henry Waldo described patients with OA experiencing a loss of power, slight sensory deficits, weakness, fatigue, and associated pain [6]. More recently with advances in technology has come the understanding of OA on a biochemical level. Many times there exists a disruption in the natural balance of cartilage degeneration from proteolytic enzymes and synthesis from anabolic growth factors [7]. Humans are the main focus of OA research but the disease is not limited to one species. OA can affect many different species with one of the oldest fossil records found in a Caudipteryx that roamed the earth more than 130 million years ago [8].

OA diagnosis and tracking

Patients suffering from OA tend to present with joint pain and reduced range of motion. The first attempts to radiographically classify OA were described by Kellgren and Lawrence in 1957 [9]. The Kellgren-Lawrence (KL) scale uses five grades and was developed after looking at eight different joints. Minimally, a grade zero shows no signs of observable radiographic changes, whereas grade four shows radiographic changes of multiple osteophytes and bone end deformities [9]. The KL grading scale is commonly used for epidemiological studies, to develop treatment protocols, and is utilized in documentation to gain approval for specific treatments [10].

OA is clinically diagnosed if a patient has usage-related joint pain in multiple joints, is over the age of 45 years, and has morning stiffness for less than 30 minutes. Because OA is a wear-and-tear disease, pain affects joints used with higher frequency and peaks during the middle of the day as usage increases. The first line imaging modality is plain radiographs and findings to diagnose OA include decreased joint spaces, formation of osteophytes, and subchondral sclerosis [11]. Other imaging modalities include computed tomography (CT), magnetic resonance imaging (MRI), and ultrasound (US). CT and MRI are often incorporated when a more detailed picture of the osseous structures and soft tissues is required. The advancements in CT and MRI have allowed physicians to find articular degeneration earlier and with more precision. However, the cost of screening and overexposure to radiation by CT must be considered. Ultrasound is another option that can be incorporated and has become more sensitive in recent years. Ultrasound has the ability to detect certain changes around a joint that suggest an early inflammatory process is occurring. Ultimately, these modalities have benefits where they should be incorporated but can also have limitations in the early diagnosis of OA before progressing to degeneration. 

Here we present a review of a proposed diagnostic algorithm for OA that also lays out current guidelines, various minimally invasive treatments and surgical options, and two screening modalities that may be incorporated to diagnose patients in a pre-OA state.

## Review

Mild versus moderate versus severe OA

**Figure 1:**
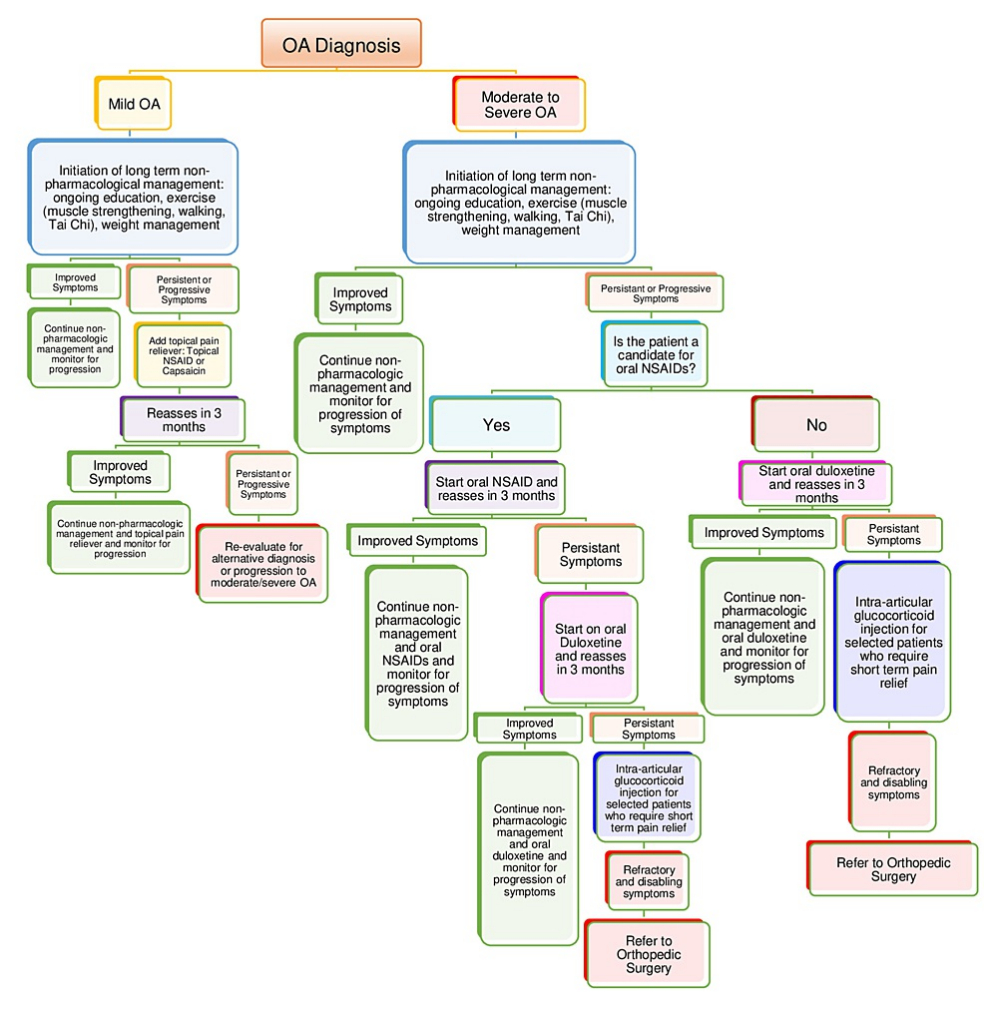
Suggested algorithm for OA management **Original Image** (OA) osteoarthritis; (NSAIDs) non-steroidal anti-inflammatory drugs

Patients with mild OA tend to have low levels of pain with preserved joint function and minimal impacts on their activities of daily living (ADLs). Those with moderate to severe OA tend to have persistent pain that significantly impacts their ADLs. Staging OA is critical to prepare a proper treatment protocol (Figure 1). Here we propose a treatment algorithm to consider for managing OA based on severity. Patients throughout both management protocols should be reassessed every three months and only continue onto a new treatment if the current one is not providing relief.

The American College of Rheumatology/Arthritis Foundation updated their guidelines in 2019 for the management of OA in the hand, hip, and knee [12]. These guidelines are broken down into two categories, strong recommendations and conditional recommendations, and those either for or against the treatment. The most important aspects are exercise and weight loss. Strong recommendations to consider include self-efficacy and self-management program, Tai chi, a cane, tibiofemoral knee braces, hand orthoses, topical non-steroidal anti-inflammatory drugs (NSAIDs), oral NSAIDs, and intra-articular glucocorticoid injections. Strong recommendations of treatments to avoid are transcutaneous electrical stimulation, bisphosphonates, glucosamine, chondroitin sulfate, hydroxychloroquine, methotrexate, platelet-rich plasma treatments, stem cell injections, tumor necrosis factor inhibitors, and interleukin-1 receptor antagonist. Conditional recommendations to consider are yoga, acupuncture, cognitive behavioral therapy, patellofemoral braces, kinesiotaping, thermal interventions, topical capsaicin, acetaminophen, duloxetine, and tramadol. Conditional treatments to avoid include modified shoes, massage therapy, pulse vibration therapy, non-tramadol opioids, colchicine, fish oil, vitamin D, intraarticular hyaluronic acid injections, intraarticular botulinum toxin injections, and prolotherapy.

Minimally invasive OA management

Intra-articular corticosteroid (IACS) injections are commonly first-line for any patient presenting with OA-related pain, however, historically they have remained controversial [13]. In some cases, IACS injections are mixed with a local anesthetic, such as lidocaine. The local anesthetic offers immediate relief while the steroid acts long-term to decrease inflammation, through immune suppression, and can provide relief for weeks to months. IACS injections are recommended as needed but no sooner than in three-month intervals, and not more than three to four times a year [13]. The first series of IACS injections often offers relief long enough that patients do not return for months at a time, but as degeneration due to OA continues, patients seek injections at shorter intervals [13].

Reports have found that IACS injections only provide short-term relief, and possibly can have negative effects on cartilage. These studies have suggested against IACS injection use as a long-term therapeutic treatment [13, 14]. Control groups in these studies only receiving saline injections seemed to have better outcomes, possibly due to the dilution of intra-articular enzymes and debris [14]. In 2000, Walker-Bone et al. supported the findings that IACS injections should not comprise the only treatment option, as it was found that multiple injections had the potential to accelerate articular damage [15]. Further research suggests against IACS injections due to toxicities and disease progression, pointing to possible dose dependency as an explanation for previously observed articular damage progression [16, 17]. Recent studies show that repeated IACS injections lead to higher KL grades due to the possible chondrotoxic properties of steroids, especially at higher doses and higher frequencies [18].

The importance of conservative management as the first-line option, such as physical therapy (PT), is key due to findings that show over a one-year period, less pain and functional disability were seen in patients compared to IACS injections [19]. Even as IACS injections have proven to be inferior to PT, IACS injections continue to be offered as a first-line treatment in many healthcare settings. Using a Medicare database, researchers showed a peak of IACS injections in newly diagnosed OA patients at around 47% in 2006, only decreasing to around 37% by 2013 [20]. The quick relief with minimal patient compliance continues to make IACS options enticing to patients and physicians.

Other intra-articular injection options include hyaluronic acid (HA), platelet-rich plasma (PRP), and mesenchymal stem cells (MSCs). HA derivative injections mimic natural synovial fluid, providing lubrication and protection to articular surfaces. However, the benefit of their use has limited evidence and they remain controversial [21]. PRP injections are a promising new technique, but there is little understanding of their mechanism of action. PRP injections were found to be superior to HA injections in relieving pain in younger patients, but not in those over 50 years of age being treated for advanced OA [21]. As cell isolation techniques have improved in recent years, MSCs have been used more frequently, but their effectiveness as a joint degeneration therapy is still debated. A recent meta-analysis found that there is limited evidence that pain relief and functional improvements for OA are achieved when administering MSC injections [22].

The American College of Rheumatology/Arthritis Foundation’s updated guidelines for managing OA in the hand, hip, and knee gave specifics regarding various joint injections [12]. IACS injections are strongly recommended as a treatment for hip and knee OA and conditionally for hand OA. The panel agreed to the insufficient data available to judge the use of short-term versus long-term preparations and choice of dose. They acknowledged the possibility of cartilage loss from these injections but were unsure of the actual clinical significance of these findings due to no associated findings of exacerbating pain, reduced function, or worsening radiological changes. HA injections were not recommended utilizing due to the available evidence having bias and failing to establish a clear benefit. The insufficient evidence is higher with respect to the hips and therefore deemed as a stronger recommendation against its use as a treatment for hip OA. According to the American College of Rheumatology/Arthritis Foundation's understanding, PRP injections lack standardization and call into question the mix of plasma-rich substances being injected into a joint. Even fewer studies have verified their use as a treatment for hand OA. Therefore, they recommended against these injections for hand, hip, or knee OA. Similar to PRP injections, MSC injections lack standardization and are not recommended as a treatment for hand, hip, or knee OA.

Topical solutions such as capsaicin have been around for many years as a non-systemic relief cream for peripheral pain. Capsaicin modulates the transmission of pain from peripheral nerves by depleting the stores of a neurotransmitter substance P at sensory nerve endings and was proven to be superior to placebo in providing relief for OA-related pain [15, 23]. Topical NSAIDs gels, such as diclofenac, work similarly to oral NSAIDs by inhibiting prostaglandin synthesis. The benefit of these topicals is the avoidance of systemic effects while delivering a high concentration of the substance to the desired site. Duloxetine is a serotonin-norepinephrine reuptake inhibitor that has been demonstrated as a pain modulator in several chronic pain conditions. Between 2009 and 2011, three separate double-blind, randomized, placebo-controlled trials showed the efficacy of duloxetine for patients with chronic OA not controlled by NSAIDs [24].

Surgical treatments for OA 

Final OA management requires referral to an orthopedic surgeon for surgical considerations in patients with moderate to severe OA who have not benefited from previous interventions. Surgical options are limited based on the specific joint. Surgery ranges in invasiveness from arthroscopic debridement to partial or total arthroplasty. During arthroscopic surgery, the joint is washed out to remove debris or crystalline products and debrided at the articular surfaces. The goal of arthroscopic surgery is to decrease pain caused by osteophytes and improve the overall range of motion [25]. A significant disadvantage of an arthroscopic procedure is the inability to address the factors initially causing their OA, such as age-related or use-related degeneration. In addition, after this procedure, patients are left with reduced synovial fluid, less cartilaginous cushion, and damaged articular surfaces [25]. In many cases, patients will ultimately need to undergo a more invasive definitive treatment, such as a partial or total arthroplasty.

Total knee arthroplasties (TKAs) and total hip arthroplasties (THAs) are surgeries used as definitive treatments for patients who have failed to have relief from previous OA interventions. Although there have been recent advances in certain surgical approaches, arthroplasty surgeries commonly involve large incisions, foreign prosthetics, and intensive recoveries [26]. Hips and knees have bilateral stresses throughout a person's lifetime, often requiring a patient to undergo bilateral TKAs or THAs. Populations around the world continue to increase with higher median ages due to better healthcare and advancements in technology. In the United States, TKAs are projected to increase from 54% in 2020 to more than 401% by 2040, and THAs from 34% in 2020 to 284% by 2040 [27]. Another consideration is the need for an arthroplasty revision, especially in younger patients. Internal prosthetics wear over time and eventually fail. Revision surgeries require longer surgery times, increased cost, complicated implant fixation, joint line reconstruction, and poor ligament functional results [28]. This further illustrates the need to delay a patient's need for an invasive treatment such as a TKA or THA, and why diagnosing OA early is paramount.

Curbing the burden of OA through early diagnosis

There currently exists no clinical method to detect early OA. Two areas have been explored experimentally to possibly stage a patient as pre-OA: fluctuations in electrical signals generated by articular joints and systemic biomarkers. Below, we review current research on each of these modalities in an effort to highlight how they might be used in the diagnosis or staging of pre-OA. This is particularly important when considering the invasiveness and high cost associated with current staging and treatment practices.

Biomarkers

**Figure 2:**
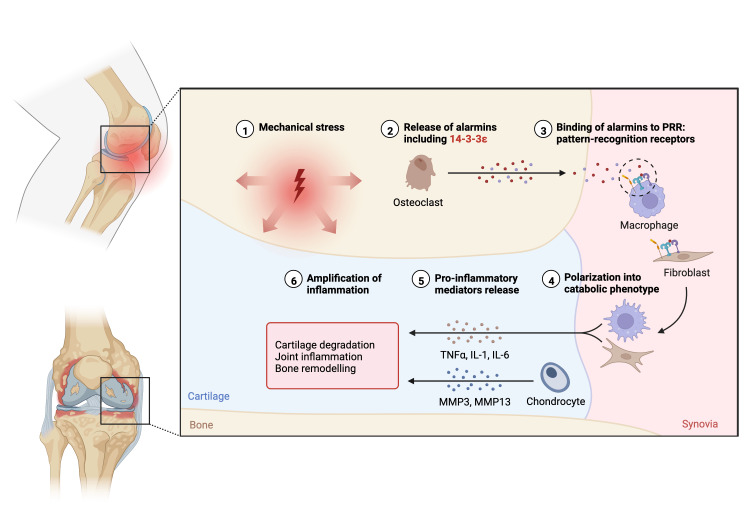
Immune cascade in osteoarthritis **Original Image created with BioRender.com** Mechanical stress (e.g., osteoarthritis-related changes) leads to the release of alarmins (e.g., 14-3-3-eta), which binds to pattern-recognition receptors (PRRs) on endothelial cells and macrophages. These cells differentiate and, along with chondrocytes, release pro-inflammatory mediators, such as interleukins (IL-1 and IL-6), tumor necrosis factor-alpha (TNF-α) and metalloproteases (MMP-3 and MMP-13). These mediators amplify the inflammatory process and lead to further cartilage destruction, joint inflammation and bone remodeling.

Biomarker levels are often screened for through routine blood draws and, if developed further, offer a cost-effective option for pre-screening, diagnosing, or staging OA. Biomarkers in circulation are under investigation as having a role in understanding the progression of OA and as tools to assess the outcomes of clinical interventions. Bauer et al. proposed the BIPED (Burden of Disease, Investigative, Prognostic, Efficacy of Intervention and Diagnostic) biomarker classification system to provide a framework to characterize OA biomarkers [29]. Promising biomarkers shown to diagnose, assess disease burden, and as a prognostic marker were urinary CTX-II (enzyme immunosorbent to determine the degradation products of c-terminal telopeptides of type 1 collagen), serum cartilage oligomeric matrix protein (COMP), and serum hyaluronan. Urinary CTX-II showed the best results as a biomarker in all three categories [29]. OA can be further understood as a disease involving low-grade systemic inflammation [30]. Common markers associated with general inflammation are c-reactive protein and various cytokines [31]. Compared to rheumatoid arthritis, where the immune response is primarily an adaptive immune response, OA is primarily an innate response (Figure 2).

The pathogenesis of OA involves the release of alarmins, such as 14-3-3-eta, that bind to pattern-recognition receptors (PRRs) on macrophages, which in turn leads to the release of proinflammatory mediators and an amplified inflammatory response [32]. Alarmins are endogenous immunomodulatory molecules that have multiple roles in activating an immune response and recruiting additional immune molecules [33]. The 14-3-3-eta biomarker protein is joint-derived and signifies an underlying inflammatory process occurring in a joint, such as increased mechanical stress in early OA patients [32]. Pro-inflammatory cytokines include interleukin-1 (IL-1), interleukin-6 (IL-6), and tumor necrosis factor-alpha (TNFα) released by endothelial cells and macrophages, and metalloproteases (MMPs) released by chondrocytes. Another biomarker that has shown promise is citrullinated protein (CP). CP was found higher in serum concentrations in early OA patients and was also found in early RA patients, but the two populations were distinguishable by levels of rheumatoid factor and/or anti-cyclic citrullinated peptide compared to CP [34]. Biomarkers are an essential systemic risk factor to consider when placing a patient in a pre-OA designation, signifying to the patient and other healthcare providers the importance of early education and disease-modifying options.

Joint electrical streaming potentials

The first electrical signals observed from joints under loading conditions were called streaming potentials [35]. The extracellular matrix of articular cartilage has collagen type II and proteoglycan aggrecans, which include glycosaminoglycans (GAG) that are charged and generate an electrical field. By adding a compressive force to the articular surface, these electrical fields produce streaming potentials. Studies show the correlation between streaming potential changes as articular cartilage changes occur. Using this information, groups developed technology to measure articular health using arthroscopic instruments by directly contacting the joint surface [36]. The issue is that this procedure is surgical and therefore invasive.

Using the idea that load-induced streaming potentials can be generated from an articular surface, authors in 2012 hypothesized that these potentials could be measured noninvasively with sensors surrounding the knee [37]. The knee proves to be an ideal joint for this technology as its joint space is close to the skin surface and under continual high stresses, so it is commonly affected by OA. The term electroarthrography (EAG) was first coined in an abstract in 2012 and later published in the journal of Osteoarthritis and Cartilage in 2013 [37]. To gather the data, known as EAG potentials, electrical sensors are attached to the medial and lateral sides of the knee in eight locations. Measurements are assessed while a patient dynamically loads the knee. Patients are asked to rock side-to-side, and this motion produces fluid movements that can be measured via an electrical monitor. These electrical potentials gathered around the knee represent the amount of healthy surface present, which represents factors such as the synovial fluid concentration and the ability of the surface to take compressive forces. Healthier knees have higher amounts of synovial fluid with low ionic strengths and can handle higher levels of compressive forces, which produces higher EAG potentials. Negative to zero electrical potentials were found in knees with a prosthetic, compared to high electrical potentials measured in a young, healthy knee [37]. This same group of authors set out to further understand this technology. Their goal was to explore factors that could affect EAG potentials. In 2016 they measured various muscle interactions and how physical activity before data collection impacted results [38,39]. They further analyzed how postural sway changes have a role in EAG potential values [40]. Muscle interactions were hypothesized to act across the knee joint, increasing the contact force across the joint [38]. EAG signals correlated well with muscle force measurements across the knee joint, proving EAG’s ability to act as a sensor to contact forces across the knee [38]. Later, this team looked at how the electromechanical ratio (EMR), which represents the produced electrical field, could be affected by physical activity prior to EAG measurements [39]. They found the EMR to be decreased in participants who had recently exercised, suggesting habituation of the stretch reflex and recommended that prior to EAG measurements, patients refrain from physical activity [39]. In 2017 the team set out to verify that dynamically loading the knee joint changes EAG potentials through postural sway [40]. They used the eight electrodes around the knee and ground-level force plates. Electromechanical models predicted EAG potentials based on ground force measurements. It was found that EAG potentials predicted based on ground force displacements correlated with measured values, reflecting those changes in contact forces through the knee through postural sway producing measurable EAG potentials [40].

The latest publication on EAG started in 2013 as an abstract, eventually coming together as a published article in the journal *Cartilage *in 2020 [41]. After coining the term, showing that potentials could be measured noninvasively, and analyzing factors that affect the values, their goal was to prove that EAG values originated from the cartilage extracellular matrix when the joint was loaded. All prior publications involved small human cohorts. To show their results, the team needed to manipulate the synovial fluid content of a joint and degrade articular surfaces. Horses can suffer from similar degenerative joint diseases as humans, and anatomical structures in horse joints are similar to those in humans. Limbs were subjected to synovial fluid replacements with differing electrolyte concentrations and the articular surfaces were degraded with trypsin [41]. Forelimbs were then hydraulically loaded, and EAG equipment was attached to the limbs at the joint space to measure values for the different experimental protocols. For this study’s results, the team calculated EAG coefficients by dividing the electrical potential measured through an EAG sensor over the force the limb was subjected to hydraulically [41]. These limbs were then dissected to assess cartilage quality directly. The results demonstrated EAG’s sensitivity to detect cartilage degeneration and synovial fluid concentrations by showing a correlation between cartilage quality or fluid concentration and calculated EAG coefficients [41]. An inverse relationship was seen between the ionic strength of synovial fluid and cartilage degeneration to EAG coefficients - as ionic strength increases and cartilage degeneration progresses, EAG coefficients decrease [41]. This same team also published an abstract in 2014 that has not been fully published as a peer-reviewed article [42]. This study used three live horses, ages seven, nine, and 16 years of age, and measured EAG coefficients as they force-loaded their forelimbs [42]. EAG coefficients were collected on all horses, and analysis of the joints showed cartilage degeneration that was not seen radiographically, demonstrating the effectiveness of EAG as a more sensitive tool in detecting early cartilage damage [42]. 

EAG is a promising new technique to detect early arthritic changes and joint degeneration before radiological findings are found [37-42]. However, the use of EAG in a clinical setting as a standard diagnostic tool has yet to be evaluated. Still, experimentally, EAG has shown to be a noninvasive and sensitive means to detect joint changes. With proper development, EAG has the potential to be a cost-effective and reproducible device for early intervention. Clinically, EAG not only shows promise in detecting early OA changes superior to radiological findings but also as a tool to evaluate the effectiveness of standard treatments and future experimental interventions.

## Conclusions

In an attempt to provide relief, OA management currently focuses on early lifestyle modifications and noninvasive treatments. Management protocols can improve by targeting modifiable risk factors such as weight loss and low-impact exercises, being more individualized, and having minimal side effects. Providing more personalized management of OA could possibly halt or slow down the progression of OA. Measurable joint damage through conventional modalities is often permanent because articular cartilage has limited healing capacity due to low metabolic activity and limited blood supply. Systemic molecular markers and superficial measured electrical joint signals, that assess early articular changes or the predisposition of an individual to future damage, may lead to an earlier diagnosis in a pre-OA state and the initiation of early management. Early staging techniques will require advancements that are cost effective for the patient with minimal adverse effects. Further research should investigate how these non-invasive modalities can be used to target OA patients before joint and articular degeneration leads to permanent damage.
